# The relationship between perceived peer motivational climate and moderate-to-vigorous physical activity in middle school physical education classes using triaxial accelerometers: an analysis of a dual-process model

**DOI:** 10.3389/fpsyg.2026.1868378

**Published:** 2026-09-04

**Authors:** Shuangyang Chu, Gyuil Lee, Jieun Yu, Wooyoung Joo

**Affiliations:** 1 Department of Physical Education, Kyungpook National University, Daegu, Republic of Korea; 2 Department of Sport and Exercise Science, Seoul Women's University, Seoul, Republic of Korea; 3 Institute of Sports Science, Pukyong National University, Busan, Republic of Korea

**Keywords:** middle school PE, MVPA, peer motivational climate, self-determined motivation, triaxial accelerometer

## Abstract

**Background:**

Adolescents’ moderate-to-vigorous physical activity (MVPA) levels are declining, and peer climate and motivational factors in physical education (PE) classes are closely associated with MVPA, but the interaction mechanisms among them remain to be further clarified. This study aimed to examine the relationship between perceived peer motivational climate and MVPA during PE classes and to test the dual-path mediating roles of autonomous and controlled motivation.

**Methods:**

Participants included 596 students (313 boys and 283 girls in grades 7–9) from five middle schools in Daegu, South Korea. MVPA levels were measured using triaxial accelerometers, while peer motivational climate and student motivation were assessed via questionnaires.

**Results:**

First, peer motivational climate, motivation, and MVPA levels differed significantly by gender and grade, with girls and higher-grade students scoring lower in task-involving climate, autonomous motivation, and MVPA. Second, a task-involving climate significantly predicted MVPA, and both autonomous motivation and controlled motivation served as mediators in this relationship. In contrast, an ego-involving climate significantly predicted MVPA, in which only controlled motivation played a mediating role, while autonomous motivation did not show a significant mediating effect. Overall, the negative effect of ego-involving climate was stronger than the positive effect of task-involving climate.

**Conclusion:**

It may be beneficial in physical education to consider girls and higher-grade students, while reducing exposure to ego-involving climates and fostering task-involving climates, which may be associated with higher levels of MVPA.

## 1 Introduction

Early adolescence is a critical period for the development of physical and psychological health. During this stage, regular physical activity (PA) not only enhances cardiorespiratory fitness and reduces the risk of cardiovascular disease but also alleviates anxiety and depression and contributes to higher self-esteem and a stronger sense of social belonging (Cai et al., 2025). In particular, moderate-to-vigorous physical activity (MVPA) provides substantial and more efficient health benefits than physical activity of other intensities over the same duration (Janssen and LeBlanc, 2010). However, numerous studies have reported that adolescents’ MVPA levels do not meet the WHO-recommended guideline of at least 60 min of MVPA per day (Beldo et al., 2020; Jussila et al., 2022). Therefore, multifaceted efforts are required to increase MVPA during adolescence, and from both public health and educational policy perspectives, there is an urgent need to develop scientifically grounded and effective intervention strategies.

MVPA primarily occurs when adolescents participate in structured physical activity programs, such as physical education (PE) classes or youth sports programs (Cheung, 2019). Research shows that on school days with PE classes, adolescents engage in significantly more MVPA than on days without PE classes (Viciana et al., 2019). Considering the important impact of PE classes on MVPA, the United States’ (US) Centers for Disease Control and Prevention advise that children (5–17 years old) should participate in MVPA for 50% of PE lesson time to gain appropriate health and academic benefits (Hollis et al., 2016). However, studies have shown that students often fail to achieve the recommended levels of MVPA during PE classes (Hollis et al., 2016; Nettlefold et al., 2011). These findings suggest that although PE classes offer an important opportunity for adolescents’ physical activity, their actual effectiveness in promoting MVPA remains limited. Therefore, identifying the factors that influence MVPA in PE classes and elucidating their underlying mechanisms holds significant theoretical and practical implications.

Adolescents’ physical activity levels have primarily been assessed using self-report questionnaires (e.g., the IPAQ, LTEQ), which are widely employed in large-scale surveys owing to their low cost and minimal participant burden (Craig et al., 2003). However, because these instruments rely on recall, they are prone to overestimating physical activity levels (Colley et al., 2019). In contrast, triaxial accelerometers can objectively and precisely record a wide range of physical activity data, including step counts, energy expenditure, and duration and proportion of activity at different intensity levels. Owing to their ease of use and simple operation, they are considered effective and reliable measurement tools (Migueles et al., 2017). Although accelerometers have limitations in capturing participation time for certain activities (e.g., cycling, skating, weight training, and swimming) (Phillips et al., 2021), these limitations can be adequately controlled in PE class settings. Therefore, the present study employs triaxial accelerometers to obtain a more objective assessment of MVPA levels during PE classes.

“Motivational climate” refers to an individual’s perceptions of motivational cues and expectations within a specific context, which shape goal orientations and engagement behaviors (Ames, 1992). According to achievement goal theory (Ames, 1992), motivational climate is typically distinguished into task involvement (or mastery orientation) and ego involvement (or performance orientation). A task-involving climate emphasizes effort, learning, and personal improvement. It treats mistakes as part of the learning process and values cooperation and support. In contrast, an ego-involving climate emphasizes social comparison and competition, focusing on demonstrating superior performance over others.

In the context of school PE, motivational climate is closely linked to adolescents’ MVPA. Deng et al. (2023), through a 16-week intervention study, reported that a PE program fostering a positive (autonomy-supportive) motivational climate significantly increased adolescents’ MVPA, with an average daily increase of approximately 12.7 min. Similarly, Yang et al. (2024) found that an intervention centered on a task-involving climate significantly enhanced middle school students’ sustained physical activity levels both during and outside PE classes, with stronger effects observed as the intervention duration increased. Notably, Kokkonen et al. (2020) demonstrated that task-involving and ego-involving motivational climates may influence MVPA through different motivational pathways: an ego-involving climate primarily affects MVPA via external motivation, whereas a task-involving climate promotes MVPA through pathways such as cooperation, intrinsic motivation, and exercise intention.

While existing studies largely focus on the relationship between motivational climates created by PE teachers (or coaches) and MVPA (Deng et al., 2023; Kokkonen et al., 2020; Yang et al., 2024), research examining the relationship between peer-created motivational climates and MVPA remains scarce. Adolescents’ physical activity behaviors are influenced by who creates the motivational climate (teachers vs. peers) (Habeeb et al., 2023). In particular, during middle school—a period corresponding to early adolescence—physical activity behaviors are more strongly influenced by peer relationships than by parents or teachers (Huang et al., 2023). Therefore, the present study explores the impact of the peer-related motivational climate on MVPA during middle school PE classes.

According to self-determination theory (SDT), human behavior is driven by the satisfaction of three basic psychological needs: autonomy, competence, and relatedness (Ryan and Deci, 2020). SDT also proposes a motivation continuum to explain how varying levels of psychological need satisfaction translate into different types of motivation. This continuum encompasses autonomous motivation, characterized by a high degree of self-determination; four types of extrinsic motivation that differ in how external pressures are internalized and interpreted; and amotivation, reflecting a lack of intention and purpose behind the behavior.

In sport and PE contexts, peer interactions significantly influence levels of self-determined motivation. A task-involving peer climate contributes to the satisfaction of autonomy, competence, and relatedness needs by emphasizing cooperation, effort, and self-improvement (Murcia et al., 2008). In contrast, an ego-involving climate, which prioritizes competition and social comparison, is more likely to lead to need frustration and is associated with controlling behaviors (Bureau et al., 2022). Prior research consistently demonstrates that self-determined motivation is a key determinant of physical activity participation. Basic psychological need satisfaction and autonomous motivation have been shown to be strongly associated with active learning behaviors in PE classes (Ntoumanis, 2005), as well as with physical activity intentions and sustained participation (Shen et al., 2007). Moreover, Chapman et al. (2024) reported that autonomous motivation correlates positively with MVPA, whereas controlled motivation is associated with lower MVPA and greater sedentary behavior. Longitudinal evidence from Fenton et al. (2021) further indicates that changes in autonomous motivation significantly predict increases in MVPA. Together, these findings suggest that in a task-involving peer motivational climate, students are more likely to develop higher autonomous motivation, which in turn leads to higher MVPA. In contrast, an ego-involving peer motivational climate tends to promote controlled motivation, which is generally associated with lower levels of MVPA. However, according to SDT, controlled motivation is not inherently negative, as it may still promote physical activity under certain circumstances. Compared with autonomous motivation, however, it represents a lower-quality and less stable form of regulation that is less likely to sustain long-term participation.

According to the dual-process motivational mediation model of SDT, social environmental factors influence behavior through two opposing psychological pathways that operate as independent processes (Haerens et al., 2015). Specifically, a task-involving climate forms a “pathway” that promotes MVPA by enhancing need satisfaction and autonomous motivation, whereas an ego-involving climate creates a “pathway” that undermines MVPA by intensifying need frustration and controlled motivation. Therefore, based on this dual-process framework, the present study examines how peer motivational climate affects students’ motivation and MVPA during PE classes and elucidates the mechanisms underlying these effects.

In adolescent physical activity research, gender and grade level are regarded as important variables. Adolescents may differ in environmental perceptions, motivational levels, and MVPA by gender and grade. Research indicates that boys generally demonstrate higher levels of MVPA than girls, and that overall MVPA among middle school students declines as grade level increases (Ross et al., 2023). From a motivational perspective, boys report higher levels of interest in exercise, self-efficacy, and autonomous motivation than girls, while motivational levels tend to decrease with increasing grade level (Janssen and LeBlanc, 2010). In addition, boys exhibit more positive achievement-related emotions and stronger task engagement intentions in PE classes (Fierro-Suero et al., 2022). However, existing studies examining the relationships among motivational climate, motivation, and MVPA in middle school PE classes using triaxial accelerometers have rarely investigated gender and grade differences simultaneously. Therefore, the present study set gender and grade as grouping variables to analyze differences among variables and as control variables to minimize potential confounding effects in interpreting the results.

Although studies have examined the relationships among peer motivational climate, motivation, and physical activity, several limitations remain. First, most studies have measured physical activity levels using self-report questionnaires, which are subject to subjective bias and struggle to accurately reflect actual exercise intensity during PE classes. Second, research examining the relationship between peer motivational climate and MVPA in the middle school PE context remains limited. Therefore, this study constructs a structural equation model, “peer motivational climate–self-determined motivation–MVPA.” The model clarifies how peer motivational climate affects middle school students’ MVPA during PE classes and examines the mediating effects of autonomous and controlled motivation.

*The study proposes the following hypotheses:*

*H1*: Male students will report higher levels of task-involving peer motivational climate, autonomous motivation, and MVPA, whereas female students will report higher levels of ego-involving peer motivational climate and controlled motivation during PE classes.

*H2*: Students in lower grades will report higher levels of task-involving peer motivational climate, autonomous motivation, and MVPA, whereas students in higher grades will report higher levels of ego-involving peer motivational climate and controlled motivation during PE classes.

*H3*: A task-involving peer motivational climate will positively predict middle school students’ MVPA levels during PE classes.

*H4*: An ego-involving peer motivational climate will negatively predict middle school students’ MVPA levels during PE classes.

*H5*: A task-involving peer motivational climate will indirectly positively predict MVPA during PE classes through enhanced autonomous motivation.

*H6*: A task-involving peer motivational climate will indirectly positively predict MVPA during PE classes through its negative association with controlled motivation.

*H7*: An ego-involving peer motivational climate will indirectly negatively predict MVPA during PE classes through reduced autonomous motivation.

*H8*: An ego-involving peer motivational climate will indirectly negatively predict MVPA during PE classes through increased controlled motivation.

## 2 Methods

**Figure 1:**
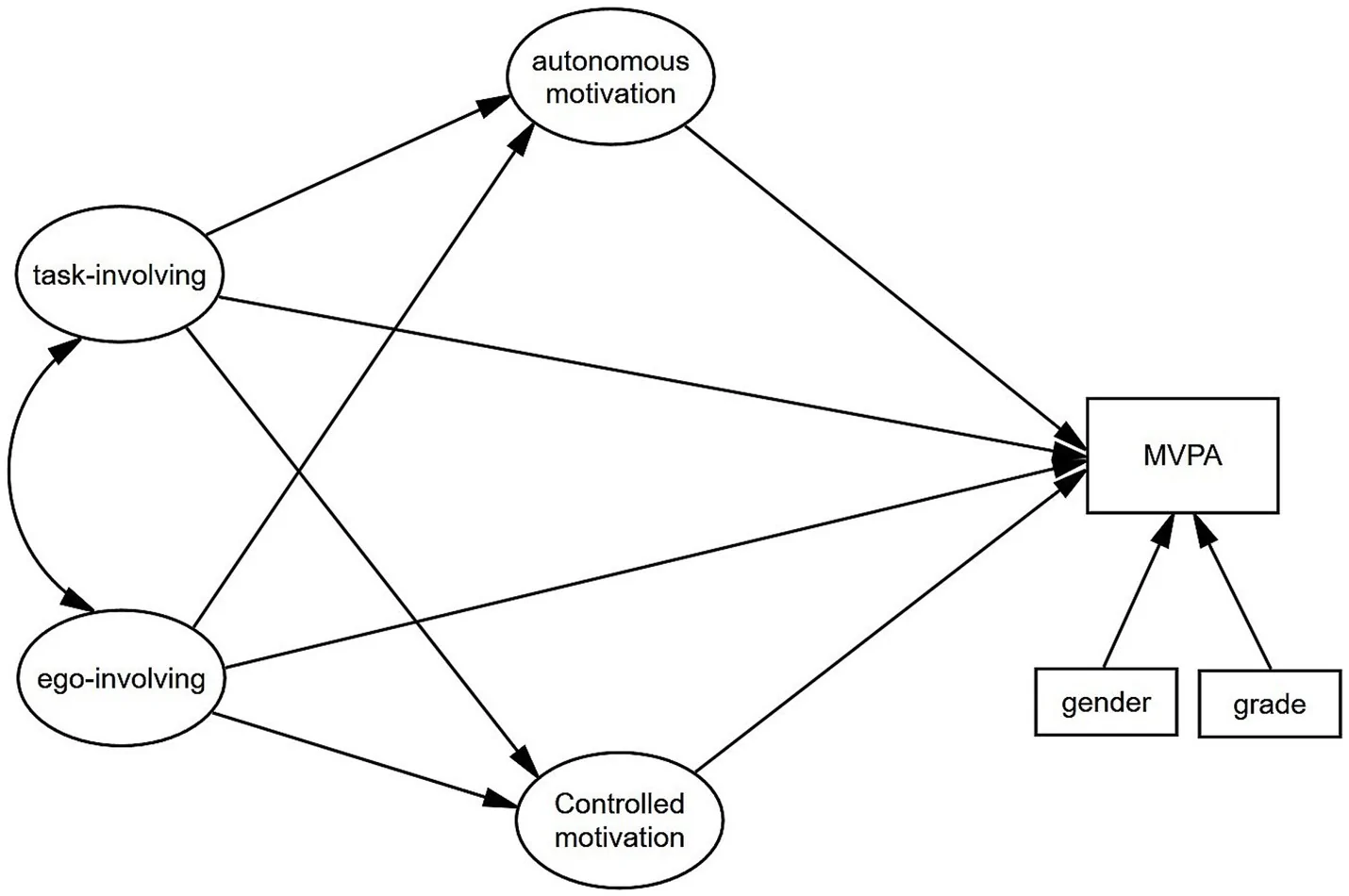
Research model. MVPA, moderate-to-vigorous physical activity.

The research model of the present study is shown in Figure 1, and the research procedures are as follows.

### 2.1 Participants

This study adopted multistage cluster sampling. Standardized inclusion and exclusion criteria were implemented throughout the sample screening process to guarantee data reliability and validity.

Stage 1 (School-level cluster sampling): Five formal coeducational middle schools in Daegu Metropolitan City delivering the unified national standardized physical education curriculum were selected to control institutional heterogeneity across schools. Physical education lessons were conducted according to each school’s pre-planned curriculum (e.g., soccer, athletics, dance, and physical fitness training) and included instructional components such as teacher instruction, individual skill practice, group practice, and appropriate small-group activities. The lessons emphasized cooperative learning and task completion, providing students with opportunities to practice and master motor skills autonomously rather than focusing primarily on competitive performance. Stage 2 (Classroom-level cluster sampling): Within each school, only intact classrooms taught by a single physical education teacher with identical curriculum progress were selected to minimize exogenous confounding attributable to individual teacher characteristics. A total of five physical education teachers participated in the study, including four male teachers and one female teacher. All teachers graduated from colleges of education with a major in physical education, held formal physical education teaching certificates, had a mean age of 33.7 years, and possessed more than 5 years of teaching experience. Stage 3 (Individual student screening): Students who were capable of completing the physical activity assessment and questionnaire and who provided written informed consent signed by both themselves and their guardians were included in the study.

The initial sample consisted of 632 students from 30 classes across five schools. The number of participating students per school ranged from 94 to 228, with an average of approximately 126 students per school. The number of participating students per class ranged from 7 to 30, with an average of approximately 21 students per class. During data cleaning, all cases were screened according to the predefined exclusion criteria. Eight participants were excluded because of insufficient accelerometer wear time or abnormal device data, and an additional 30 participants were excluded because of careless questionnaire responses or substantial missing data. The final analytic sample comprised 596 students, yielding a valid response rate of 94.3%. The final sample included 271 seventh-grade students, 124 eighth-grade students, and 201 ninth-grade students, including 313 boys and 283 girls, with a mean age of 13.9 years.

### 2.2 Measures

#### 2.2.1 Triaxial accelerometer

Participants’ MVPA levels during PE classes(45 min) were measured using a triaxial accelerometer (ActiGraph GT3X model). The device measures movement along three axes—anteroposterior, mediolateral, and vertical—through built-in sensors. To remove acceleration signals that do not correspond to human movement, a band-pass filter was applied with the detection frequency range set to 0.25–2.5 Hz (Migueles et al., 2017). The measurement duration was uniformly set to 45 min, with a standardized sampling frequency of 30 Hz. Because adolescents’ physical activity often occurs within short time intervals, the epoch length was set to 10 s (Migueles et al., 2017). Subsequently, activity intensity was classified using the criteria proposed by Evenson et al. (2008): sedentary (≤50 counts), light (51–1,148 counts), moderate (1149–2005 counts), and vigorous (≥2006 counts). These cut points are regarded as among the most valid methods for assessing physical activity intensity in children and adolescents (Migueles et al., 2017). Furthermore, to ensure data validity, records with counts per minute (CPM) of zero or less for 20 consecutive minutes or longer were excluded from the analysis (Esliger et al., 2005).

#### 2.2.2 Peer motivational climate scale

Peer motivational climate was assessed using the scale developed by Ntoumanis and Vazou (2005), which was translated and validated for the Korean educational context by Cheon and Song (2011). The original scale comprises 21 items and consists of two factors: a task-involving peer motivational climate (12 items; e.g., “recognize teammates,” “encourage teammates”) and an ego-involving peer motivational climate (9 items; e.g., “criticize teammates,” “mock teammates”). Responses were recorded on a 7-point Likert scale (1 = “strongly disagree,” 7 = “strongly agree”), with higher scores indicating a stronger perception of the corresponding type of motivational climate.

Preliminary confirmatory factor analysis (CFA) revealed two problematic items with suboptimal factor loadings and considerable error covariance. The standardized factor loading of the task-involving item “help other friends improve” was 0.462, below the ideal cutoff of 0.5, accompanied by a maximum modification index of 128.7, which suggested severe error correlations with other scale items. The ego-involving item “criticize other friends when they make mistakes” showed a standardized loading of 0.449 (also under 0.5) and a maximum modification index of 139.3, pointing to prominent cross-item error associations. Following the scale revision principles proposed by Worthington and Whittaker (2006), we retained the original two-factor theoretical structure while comprehensively weighing item loadings and CFA modification indices to decide item deletion. From a content validity perspective, the wording of these two items overlapped moderately with other entries in the scale. After removing the two items, the two-factor structure remained stable, and overall model fit indices were improved to an acceptable range, verifying that the construct validity of the scale was not substantially compromised. The final scale consisted of 19 items (11 task-involving and 8 ego-involving). Factor scores were calculated as the mean of the corresponding items. The scale demonstrated acceptable reliability (task-involving *α* = 0.956; ego-involving *α* = 0.969). Furthermore, CFA indicated an acceptable model fit (*χ*^2^ = 415.527, df = 151, *χ*^2^/df = 2.752, RMSEA = 0.054, GFI = 0.931, NFI = 0.965, CFI = 0.977, IFI = 0.977).

#### 2.2.3 Motivation

Motivation was assessed using four subscales of the Perceived Locus of Causality Scale—intrinsic motivation, identified regulation, introjected regulation, and external regulation—originally developed by Vlachopoulos et al. (2011) and translated and validated for the Korean physical education context by Song and Cheon (2014). Following Viksi and Tilga (2022), intrinsic motivation and identified regulation were combined to represent autonomous motivation, whereas introjected regulation and external regulation were combined to represent controlled motivation. The autonomous motivation subscale consisted of eight items (e.g., “because physical education is fun” and “because physical education is important to me”), and controlled motivation consisted of seven items (e.g., “to be seen as a good student by the teacher” and “because I might get scolded if I do not participate”). Responses were recorded on a 7-point Likert scale, with higher scores indicating higher levels of the corresponding motivation type. The subscales demonstrated excellent reliability (autonomous motivation *α* = 0.941; controlled motivation α = 0.935). CFA also demonstrated an acceptable model fit (*χ*^2^ = 331.466, df = 89, *χ*^2^/df = 3.724, RMSEA = 0.068, GFI = 0.926, NFI = 0.954, CFI = 0.966, IFI = 0.966).

### 2.3 Procedure

This study was conducted from March to May 2026. After obtaining consent from school principals, students, and their guardians, questionnaires and physical activity measurements were administered during PE classes with the assistance of PE teachers. The questionnaires were completed after the PE teachers explained each item and ensured that students fully understood the content. Because PE schedules varied across schools, data collection was conducted on different dates at the five participating schools. However, each participant’s accelerometer data were collected only once during a single 45-min PE lesson on one measurement day. To ensure measurement efficiency, participant information (student ID number, gender, height, weight, and age) was collected 3 days prior to measurement, and the information for the first batch of participants was entered into the devices in advance. On the day of data collection, students were instructed to wear the accelerometer on their waists. This placement was simple and convenient and did not interfere with students’ normal physical activities. Students were also informed that they could discontinue participation at any time if they experienced any discomfort during the measurement. Before the measurement began, the research team checked the placement of each accelerometer individually to ensure that it was worn correctly. During the measurement, the research team continuously monitored the placement of the accelerometers and reminded students to reposition the devices immediately if they became detached. After each PE class, the devices were collected immediately and the data were downloaded. The information for the next batch of participants was then entered into the devices, and identical measurement settings were applied to prepare the accelerometers for the subsequent class.

### 2.4 Statistical analysis

Data were analyzed using SPSS 29.0 and AMOS 28.0. After data screening, SPSS was used to examine the internal consistency of the questionnaire items, and confirmatory factor analysis was conducted using AMOS. Descriptive statistics and group difference analyses for all variables were performed according to gender and grade. Pearson correlation analyses were then conducted to examine the relationships among task-involving and ego-involving peer motivational climate, autonomous motivation, controlled motivation, and MVPA. Finally, structural equation modeling was performed using AMOS 28.0 to test whether autonomous and controlled motivation mediated the relationship between peer motivational climate and MVPA, and the effects of each path were estimated.

## 3 Results

### 3.1 Descriptive statistics of the variables

To examine configural, metric, and scalar invariance sequentially, this study performed multiple-group analysis on the peer motivational climate scale and motivation scale, with gender and grade treated as grouping variables, respectively. The results revealed that the Δ_CFI_ values between nested models across gender and grade groups were all less than 0.01, supporting scalar invariance for both scales. This finding indicates that the measurement properties of the two scales are stable across different gender and grade subgroups, which provides a valid statistical foundation for the subsequent latent mean comparisons across groups proposed in Hypotheses 1 and 2.

As shown in Table 1, significant differences were found among the variables by gender. Boys reported significantly higher scores than girls in task-involving peer motivational climate (*t* = 4.211, *p* < 0.001), autonomous motivation (*t* = 2.837, *p* < 0.01), and MVPA (*t* = 9.743, *p* < 0.001). In contrast, girls demonstrated significantly higher scores in ego-involving peer motivational climate (*t* = −4.760, *p* < 0.001) and controlled motivation (*t* = −6.153, *p* < 0.001). One-way analysis of variance (ANOVA) examining grade-level differences revealed significant variations in task-involving peer motivational climate (*F* = 11.759, *p* < 0.001), ego-involving peer motivational climate (*F* = 7.838, *p* < 0.001), autonomous motivation (*F* = 8.520, *p* < 0.001), controlled motivation (*F* = 20.975, *p* < 0.001), and MVPA (*F* = 23.630, *p* < 0.001). *Post hoc* analyses showed that ninth-grade students reported significantly lower levels of task-involving peer motivational climate and autonomous motivation than seventh- and eighth-grade students. On the other hand, ego-involving peer motivational climate was significantly lower among seventh-grade students than among ninth-grade students. Controlled motivation showed a significant upward trend across grades (Grade 7 < Grade 8 < Grade 9). MVPA levels were significantly higher among seventh-grade students, followed by eighth-grade students, and lowest among ninth-grade students (Grade 7 > Grade 8 > Grade 9).

**Table 1:** Descriptive statistics (*M* ± SD) and group differences in study variables.

Group	Number	Task-involving	Ego-involving	Autonomous motivation	Controlled motivation	MVPA
Boys	313	4.2 ± 1.6 (1.2, 6.9)	3.5 ± 1.5 (1, 7)	4.1 ± 1.4 (1.1, 6.9)	3.0 ± 1.2 (1, 6.7)	13.6 ± 6.1 (2.2, 33.8)
Girls	283	3.7 ± 1.5 (1.1, 6.9)	4.1 ± 1.7 (1, 7)	3.8 ± 1.1 (1.3, 6.8)	3.7 ± 1.5 (1.1, 6.9)	9.3 ± 4.7 (1.3, 29.2)
T		4.211***	−4.760***	2.837**	−6.153***	9.743***
Grade 7	271	4.2 ± 1.6 (1.2, 6.7)	3.5 ± 1.6 (1, 7)	4.2 ± 1.4 (1.1, 6.9)	3.0 ± 1.2 (1.1, 6.7)	13.1 ± 6.5 (2.2, 33.8)
Grade 8	124	4.0 ± 1.7 (1.2, 6.9)	3.9 ± 1.7 (1, 6.9)	4.0 ± 1.3 (1.3, 6.9)	3.4 ± 1.4 (1.1, 6.7)	11.5 ± 6.2 (2.8, 27.5)
Grade 9	201	3.5 ± 1.4 (1.1, 6.9)	4.1 ± 1.6 (1, 7)	3.7 ± 1.0 (1.4, 6.6)	3.8 ± 1.4 (1, 6.9)	9.5 ± 4.0 (1.3, 19.5)
F		11.759***	7.838***	8.520***	20.975***	23.630***
Total	596	3.9 ± 1.6 (1.1, 6.9)	3.8 ± 1.6 (1, 7)	4.0 ± 1.3 (1.1, 6.9)	3.4 ± 1.4 (1, 6.9)	11.5 ± 5.9 (1.3, 33.8)

MVPA, moderate-to-vigorous physical activity; values in parentheses indicate the minimum and maximum values; **p < 0.01, ***p < 0.001.

### 3.2 Correlation analysis

Table 2 presents the correlation analysis results among peer motivational climate, exercise motivation, and MVPA. Regarding peer motivational climate, task-involving and ego-involving peer climates showed a significant negative correlation (*r* = −0.484, *p* < 0.01), indicating that an environment emphasizing effort, cooperation, and improvement is essentially opposed to a climate that emphasizes comparison and competition. For the relationships between peer motivational climate and student motivation, task-involving peer climate was significantly positively correlated with autonomous motivation (*r* = 0.548, *p* < 0.01) and significantly negatively correlated with controlled motivation (*r* = −0.436, *p* < 0.01). In contrast, an ego-involving peer climate showed a significant negative correlation with autonomous motivation (*r* = −0.288, *p* < 0.01) and a significant positive correlation with controlled motivation (*r* = 0.586, *p* < 0.01). Additionally, a significant negative correlation was found between autonomous and controlled motivation (*r* = −0.289, *p* < 0.01).

**Table 2:** Statistics of Pearson correlation coefficient.

Variable	1	2	3	4	5
1. Task-involving	1				
2. Ego-involving	−0.484**	1			
3. Autonomous motivation	0.548**	−0.288**	1		
4. Controlled motivation	−0.436**	0.586**	−0.289**	1	
5. MVPA	0.547**	−0.611**	0.402**	−0.524**	1

MVPA, moderate-to-vigorous physical activity; **p < 0.01, ***p < 0.001.

Regarding the relationship between exercise motivation and physical activity, autonomous motivation was significantly positively correlated with MVPA (*r* = 0.402, *p* < 0.01), whereas controlled motivation was significantly negatively correlated with MVPA (*r* = −0.524, *p* < 0.01). Additionally, MVPA showed a significant positive correlation with task-involving peer climate (*r* = 0.547, *p* < 0.01) and a significant negative correlation with ego-involving peer climate (*r* = −0.611, *p* < 0.01).

Overall, the results indicated that a task-involving peer climate is associated with higher autonomous motivation and higher MVPA, whereas an ego-involving peer climate is associated with controlled motivation and lower MVPA.

### 3.3 Structural equation model

#### 3.3.1 Model fit

**Figure 2:**
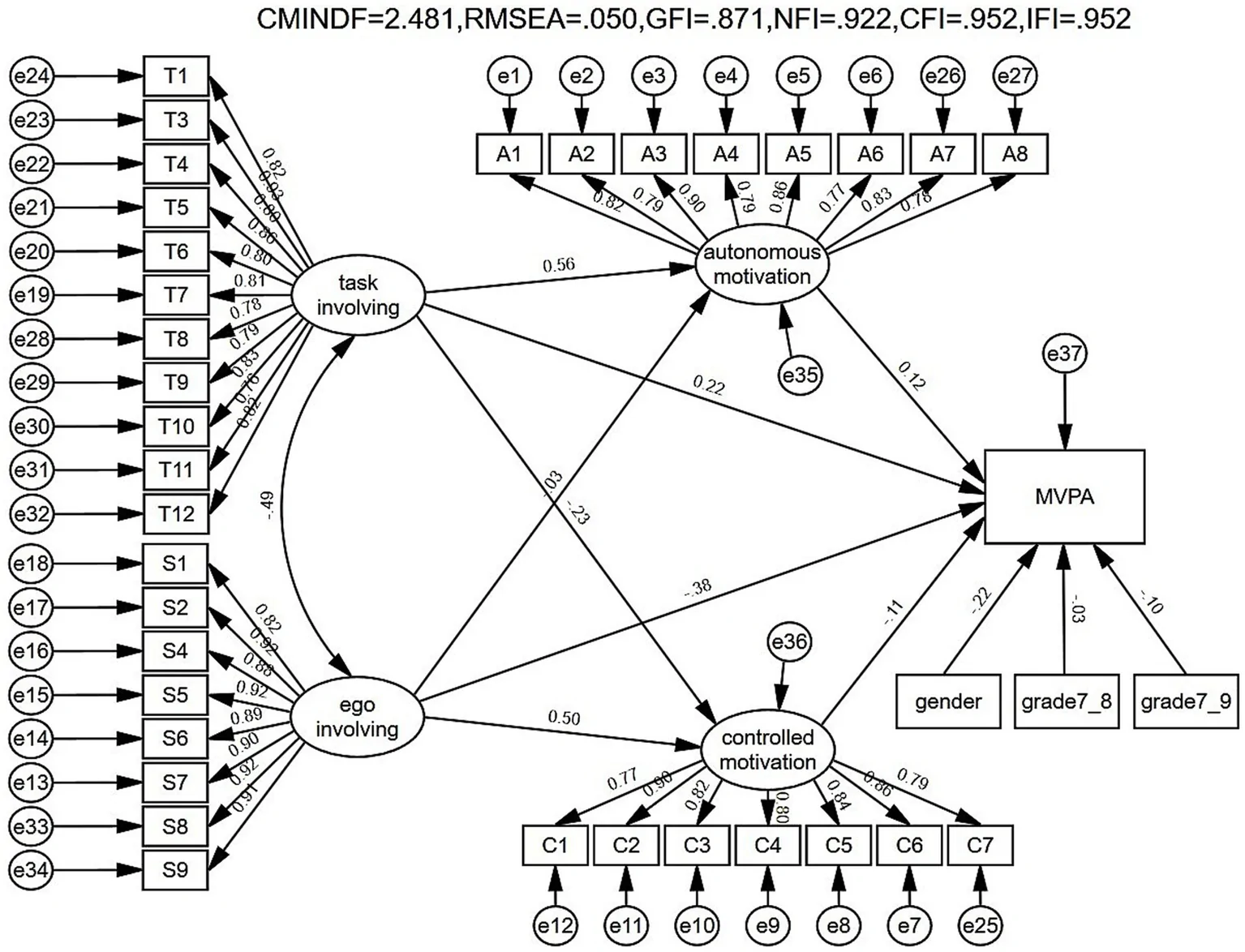
Mediation effect model. MVPA, moderate-to-vigorous physical activity.

A structural equation model was constructed to examine the relationships between task-involving peer motivational climate, ego-involving peer motivational climate, autonomous motivation, controlled motivation, and MVPA (Figure 2). Based on the difference analyses, gender and grade were included as control variables for MVPA. The fit indices of the final model were as follows: Chi-square = 1630.218 (*p* < 0.001), df = 657, *χ*^2^/df = 2.481, RMSEA = 0.050, GFI = 0.871, NFI = 0.922, CFI = 0.952, IFI = 0.952. All indices fell within the acceptable range, indicating an acceptable model fit.

#### 3.3.2 Path analysis results

As shown in Figure 2 and Table 3, most structural paths among the main variables were statistically significant after controlling for gender and grade. Task-involving peer motivational climate was significantly and positively associated with autonomous motivation (*β* = 0.563, *p* < 0.001) and negatively associated with controlled motivation (*β* = −0.23, *p* < 0.001). In contrast, an ego-involving peer climate was not significantly associated with autonomous motivation (*β* = −0.033, *p* > 0.05), but was positively associated with controlled motivation (*β* = 0.502, *p* < 0.001). Regarding the associations between motivation and physical activity, autonomous motivation was significantly and positively associated with MVPA (*β* = 0.115, *p* < 0.01), whereas controlled motivation was significantly and negatively associated with MVPA (*β* = −0.111, *p* < 0.01). Task-involving peer climate was also significantly and positively associated with MVPA (*β* = 0.223, *p* < 0.001), while ego-involving peer climate was significantly and negatively associated with MVPA (*β* = −0.382, *p* < 0.001). Concerning control variables, being female was significantly and negatively associated with MVPA compared to male students (*β* = −0.219, *p* < 0.001). Compared to the 7th grade (reference group), 8th grade was not significantly associated with MVPA (*β* = −0.027, *p* > 0.05), whereas 9th grade was significantly and negatively associated with MVPA (*β* = −0.101, *p* < 0.001).

**Table 3:** Path analysis results of the structural equation model.

Path	Standardized effect	S.E.	C.R.	*p*
Task-involving → autonomous motivation	0.563	0.031	11.864	***
Task-involving → controlled motivation	−0.23	0.03	−5.75	***
Ego-involving → autonomous motivation	−0.033	0.032	−0.8	0.424
Ego-involving → controlled motivation	0.502	0.036	12.029	***
Autonomous motivation → MVPA	0.115	0.17	3.054	**
Controlled motivation → MVPA	−0.111	0.162	−2.751	**
Task-involving → MVPA	0.223	0.126	5.251	***
Ego-involving → MVPA	−0.382	0.142	−9.363	***
gender → MVPA	−0.219	0.327	−7.51	***
grade7_8 → MVPA	−0.027	0.403	−0.912	0.362
grade7_9 → MVPA	−0.101	0.346	−3.457	***

MVPA, moderate-to-vigorous physical activity; **p < 0.01, ***p < 0.001.

#### 3.3.3 Mediation analysis

Mediation effects were examined using the bias-corrected nonparametric percentile bootstrap method with 5,000 iterations. As shown in Table 4, the indirect effect for the pathway “task-involving peer climate → autonomous motivation → MVPA” was significant [*β* = 0.192, 95% CI (0.033, 0.377)], since the interval did not include zero. Similarly, the indirect effect for the pathway “task-involving peer climate → controlled motivation → MVPA” was 0.076 [95% CI (0.02, 0.162)], demonstrating a significant mediation effect. In contrast, the indirect effect for the pathway “ego-involving peer climate → autonomous motivation → MVPA” was not significant [*β* = −0.013, 95% CI (−0.071, 0.018)] since the interval included zero. However, the indirect effect for the pathway “ego-involving peer climate → controlled motivation → MVPA” was significant [*β* = −0.196, 95% CI (−0.366, −0.057)]. Regarding direct effects, the direct effect of task-involving peer climate on MVPA was 0.66 [95% CI (0.371, 0.942)], which was significant, and the direct effect of ego-involving peer climate on MVPA was −1.333 [95% CI (−1.638, −1.039)], also significant. The total effect of the model was −0.614 [95% CI (−1.018, −0.19)], which did not include zero, indicating overall significance.

**Table 4:** Mediating effects and effect sizes (Bootstrap = 5,000).

Parameter	Path relationship	Effect	Boot95%CI	
			Lower	Upper
Indirect effect 1	Task-involving → autonomous motivation → MVPA	0.192	0.033	0.377
Indirect effect 2	Task-involving → controlled motivation → MVPA	0.076	0.02	0.162
Indirect effect 3	Ego-involving → autonomous motivation → MVPA	−0.013	−0.071	0.018
Indirect effect 4	Ego-involving → controlled motivation → MVPA	−0.196	−0.366	−0.057
Direct effect 1	Task-involving → MVPA	0.66	0.371	0.942
Direct effect 2	Ego-involving → MVPA	−1.333	−1.638	−1.039
Total effect		−0.614	−1.018	−0.19

## 4 Discussion

This study, drawing on Achievement Goal Theory (AGT) and Self-Determination Theory (SDT), examined the structural relationships among peer motivational climate (task- and ego-involving), exercise motivation (autonomous and controlled), and MVPA in PE classes using a dual-pathway mediation model. Additionally, meaningful insights were obtained through analyses of mean differences in the study variables by gender and grade level.

### 4.1 Analysis of differences: discussion of hypotheses 1 and 2

Regarding gender differences, male students showed significantly higher levels of task-involving peer climate, autonomous motivation, and MVPA, whereas female students scored significantly higher on ego-involving peer climate and controlled motivation, thereby supporting Hypothesis 1.

Boys’ higher task-involving climate scores may be attributable to their greater likelihood of experiencing positive peer interactions during PE classes. Influenced by traditional gender-role norms in East Asian societies (Xu et al., 2021), boys are generally more likely than girls to actively participate in sports. Combined with their generally higher levels of physical activity (Janssen and LeBlanc, 2010), boys are more likely to assume dominant roles, experience success, and receive recognition and support from their peers in PE classes that primarily involve ball games and team-based activities (Rodrigues et al., 2024). Consequently, boys may be more likely to perceive a task-involving peer motivational climate that emphasizes cooperation, effort, and shared improvement. In contrast, girls’ higher perceptions of an ego-involving peer climate may reflect their greater sensitivity to social evaluation and peer comparison. Adolescent girls generally exhibit higher levels of evaluation anxiety, body consciousness, and sensitivity to peer relationships (Sabiston et al., 2019). As a result, in PE settings that require the public display of physical competence, they may be more likely to focus on peer comparison, evaluation, and even negative feedback. Consequently, even when exposed to the same classroom environment, girls may interpret peer interactions as being more competitive and comparison-oriented, leading to stronger perceptions of an ego-involving peer motivational climate. Furthermore, boys generally report higher levels of exercise self-efficacy than girls (Cengız and Tılmac, 2018). Higher exercise self-efficacy enhances individuals’ confidence in their physical abilities, thereby fostering greater intrinsic motivation and a stronger willingness to engage in physical activity (Tian et al., 2026), which may partly explain the observed gender differences in motivation. Gender differences in MVPA may also be jointly influenced by sociocultural, psychological, and physiological factors. Sociocultural gender stereotypes may provide boys with greater opportunities and support for participation in physical activity (Xu et al., 2021), while higher exercise self-efficacy further strengthens their confidence in maintaining regular physical activity (Tian et al., 2026). In addition, adolescent boys generally possess greater muscular strength (Gillen et al., 2019) and superior cardiorespiratory fitness (e.g., VO₂max) (Armstrong and Welsman, 2019), enabling them to participate in and sustain moderate-to-vigorous physical activity more easily, which may ultimately contribute to their higher levels of MVPA.

Significant differences were observed across all grades (7th, 8th, and 9th) regarding motivational climate, motivation type, and MVPA, supporting Hypothesis 2. Post-hoc analyses showed that lower-grade students reported significantly higher scores for task-involving peer climate, autonomous motivation, and MVPA than their higher-grade counterparts, whereas higher-grade students scored higher on ego-involving peer climate and controlled motivation. With the progression of adolescence, students’ cognitive and social cognitive abilities continue to develop (Somerville, 2013), leading to a more complex understanding of classroom environments and peer interactions. As a result, they may become more attentive to competence performance and social comparison, and therefore may be more likely to perceive an ego-involving peer climate compared with younger students. Meanwhile, as grade level increases, the instructional goals, evaluation approaches, and teacher feedback strategies in physical education classes may also change. For example, classroom environments that emphasize sports skill performance, learning outcome evaluation, and ability comparison may enhance students’ perceptions of an ego-involving peer climate (Ntoumanis, 2005). Furthermore, with increasing grade level, students experience greater academic demands and examination-related pressure (Beck et al., 2024), which may not only reduce the time available for physical activity but also weaken their autonomous motivation for engaging in physical activity, ultimately contributing to lower MVPA levels. Therefore, the grade-related differences observed in this study may reflect the combined influence of adolescent development, physical education classroom contexts, and academic pressures.

In summary, gender and grade level emerged as significant factors influencing students’ MVPA levels. This emphasizes the importance of differentiated interventions in educational practice, highlighting that female and higher-grade students may be vulnerable groups regarding MVPA participation.

### 4.2 Analysis of direct effects: discussion of hypotheses 3 and 4

The structural equation modeling results showed that a task-involving peer climate significantly and positively predicted MVPA (*β* = 0.223, direct effect = 0.66, *p* < 0.001), whereas an ego-involving peer climate significantly and negatively predicted MVPA (*β* = −0.382, direct effect = −1.333, *p* < 0.001), thereby supporting Hypotheses 3 and 4. These findings are consistent with prior research (Chen et al., 2020; Deng et al., 2023; Yang et al., 2024).

A task-involving peer climate emphasizes cooperation, effort, and individual skill improvement. It encourages mutual support and collaborative task engagement, with a focus on personal growth rather than peer comparison. In this environment, students are more likely to become deeply engaged in activities and sustain activity intensity and persistence (Ntoumanis and Vazou, 2005; Shen et al., 2007), resulting in higher MVPA levels. Moreover, a task-involving climate enhances students’ willingness to participate in physical activity and boosts their self-efficacy (Ntoumanis, 2005), leading to higher physical activity levels both during and outside of class.

In contrast, an ego-involving peer climate emphasizes competition, ability comparison, and normative evaluation, which can trigger anxiety, stress, and maladaptive behavioral strategies in peer interactions (Pensgaard and Roberts, 2000). This climate may lead students to avoid or participate passively in physical activity, thereby reducing MVPA. When social comparison and competition dominate, student engagement in both class and extracurricular activities tends to decrease (Castro-Sánchez et al., 2019).

### 4.3 Analysis of mediating effects: discussion of hypotheses 5, 6, 7, and 8

The structural equation model and bootstrap mediation analysis indicated that a task-involving peer climate significantly and positively predicted autonomous motivation (*β* = 0.563, *p* < 0.001) and significantly negatively predicted controlled motivation (*β* = −0.23, *p* < 0.001). Further mediation analysis revealed that the indirect effect of a task-involving peer climate on MVPA via increased autonomous motivation was 0.192 [95% CI (0.033, 0.377), *p* < 0.05], and the indirect effect via suppression of controlled motivation was 0.076 [95% CI (0.02, 0.162), *p* < 0.01]. This finding suggests that a task-involving peer climate is associated with higher autonomous motivation, which is in turn associated with higher physical activity participation, while it is also associated with lower controlled motivation, which is related to higher MVPA levels. These findings provide support for Hypotheses 5 and 6.

A task-involving peer climate emphasizes self-improvement, cooperation, and effort rather than social comparison or competition, thereby satisfying the three basic psychological needs of autonomy, competence, and relatedness (Murcia et al., 2008). In such an environment, students perceive physical activity as an opportunity for personal growth and skill development rather than as a means of external evaluation, which in turn enhances autonomous motivation. Students with higher levels of autonomous motivation demonstrate more active participation behaviors in class, stronger intrinsic interest, and greater voluntary engagement, ultimately exhibiting higher levels of MVPA (Deng et al., 2023; Ntoumanis, 2005; Shen et al., 2007). It is noteworthy that although the indirect effect through controlled motivation was statistically significant, its effect size was relatively small. This finding may be related to the unique characteristics of physical education classes. Unlike self-selected physical activities, school-based physical education classes involve certain curricular requirements, and students are expected to participate in physical activities according to the instructional arrangements. Therefore, even when students have lower levels of autonomous motivation, they may still maintain a certain level of participation due to external factors such as course requirements, teacher expectations, or performance evaluations. According to Self-Determination Theory (SDT), although controlled motivation represents a lower-quality and less stable form of behavioral regulation compared with autonomous motivation, introjected regulation may still facilitate behavioral engagement in the short term under specific circumstances (Vasconcellos et al., 2020). Therefore, the relatively small mediating effect of controlled motivation observed in this study may reflect the influence of external demands on students’ immediate participation behaviors within the context of physical education classes.

In the negative mediation pathway analysis, the results showed that an ego-involving peer climate had a significant indirect effect on MVPA through controlled motivation [*β* = −0.196, 95% CI (−0.366, −0.057), *p* = 0.007], whereas the indirect effect through autonomous motivation was not significant. Therefore, Hypothesis 8 was supported, while Hypothesis 7 was not supported. Specifically, an ego-involving peer climate significantly and positively predicted controlled motivation (*β* = 0.502, *p* < 0.001), and reduced students’ MVPA levels by enhancing controlled motivation, indicating that its effect primarily operates through an externally pressured and anxiety-driven regulatory pathway. In contrast, the relationship between an ego-involving peer climate and autonomous motivation was not significant. This may be because autonomous motivation primarily originates from the satisfaction of basic psychological needs, a process that is more strongly supported by task-involving and autonomy-supportive environments (Murcia et al., 2008). By contrast, ego-involving peer climates are more self-centered and emphasize competition and social comparison, which are more likely to activate controlling regulatory mechanisms rather than directly undermine autonomous motivation (Bureau et al., 2022). Therefore, this type of peer climate is mainly reflected in the controlled motivation pathway rather than in a significant association with autonomous motivation, which may explain why the indirect effect of the “ego-involving peer climate → autonomous motivation → MVPA” pathway was not statistically significant.

Overall, task- and ego-involving peer climates were associated with MVPA through different pathways, showing a complex pattern of relationships, with a total effect of −0.614 [95% CI (−1.018, −0.19), *p* < 0.01]. This suggests that the negative association between ego-involving peer climate and MVPA may be stronger than the positive association between task-involving peer climate and MVPA. Therefore, in PE settings, fostering task-involving peer climates while reducing ego-involving peer climates may be important considerations for supporting adolescents’ MVPA participation. These findings provide further support for the dual-process motivational framework proposed by Self-Determination Theory and highlight the importance of considering peer-related social environments when understanding students’ physical activity participation.

Although this study provides meaningful insights into the motivational mechanisms linking peer environments to adolescents’ MVPA, several limitations should be acknowledged. First, the peer motivational climate scale used in this study primarily reflects students’ perceptions of peer interactions (e.g., encouragement, recognition, criticism, and ridicule) rather than directly assessing the broader motivational climate itself. Future studies should adopt more comprehensive measures to further clarify how different social environments influence students’ motivation and MVPA. Second, this study did not include teacher-created motivational climate, although teachers’ instructional strategies and feedback styles may also influence students’ motivation and MVPA. Future research should incorporate both teacher- and peer-related motivational climates to provide a more comprehensive understanding of PE environments. Third, MVPA was assessed during a single PE class, limiting the ability to determine long-term behavioral changes. Future longitudinal studies are needed to further examine the dynamic relationships among motivational climate, motivation, and MVPA. Nevertheless, the objective assessment of MVPA using triaxial accelerometers and the inclusion of a large sample from five schools represent important strengths of this study.

## 5 Conclusion

This study examined the relationships among peer motivational climate, motivation, and MVPA in PE classes among middle school students based on Achievement Goal Theory (AGT) and Self-Determination Theory (SDT). The findings showed that gender and grade differences existed in students’ perceptions of peer motivational climate, motivation, and MVPA, with female students and higher-grade students generally demonstrating lower levels of autonomous motivation and MVPA. Furthermore, task-involving peer climate was positively associated with MVPA, whereas ego-involving peer climate was negatively associated with MVPA. Autonomous motivation mediated the relationship between task-involving peer climate and MVPA, while controlled motivation mediated both task- and ego-involving peer climate pathways. Overall, these findings highlight the distinct motivational pathways through which peer environments are associated with adolescents’ physical activity participation. From a practical perspective, these findings suggest that PE teachers and schools should consider creating supportive peer environments that emphasize cooperation, effort, and individual improvement while reducing excessive competition and social comparison. Such approaches may help foster more positive motivational experiences and encourage greater participation in physical activity, particularly among students who are less engaged in PE classes.

## Data Availability

The original contributions presented in the study are included in the article/Supplementary material, further inquiries can be directed to the corresponding author.
